# Infliximab rescue therapy in a patient with acute severe ulcerative colitis and coronavirus disease 2019 followed by *Escherichia coli* 0157:H7 infection: a case report

**DOI:** 10.3325/cmj.2021.62.634

**Published:** 2021-12

**Authors:** Dinko Bekić, Željka Belošić Halle

**Affiliations:** Department of Gastroenterology and Hepatology, Sveti Duh University Hospital, Zagreb, Croatia

## Abstract

The management of patients with acute severe ulcerative colitis and SARS-CoV-2 presents a clinical challenge. We report on the first case of a patient with acute severe ulcerative colitis and mild coronavirus disease 2019 (COVID-19) who received rescue infliximab therapy, followed by a relapse caused by enterohemorrhagic *Escherichia coli* 0157:H7. The treatment challenges we faced were biologic therapy administration during active COVID-19, about which little was known at the time, and how to treat EHEC due to the risk of hemolytic uremic syndrome. Acute severe ulcerative colitis was treated with rescue infliximab therapy, and enteric infection with an antibiotic, both with satisfactory clinical response. The decision to induce biologic therapy for inflammatory bowel disease relapse in SARS-CoV-2-positive patients should be made on a case-to-case basis and should be driven by the dominant disease. Our patient tested positive for SARS-CoV-2, but actually had mild disease. At the same time, she had acute severe ulcerative colitis, so we started anti-tumor necrosis factor therapy despite serological tests and the recommendation to delay biological therapy administration for two-weeks. Second, due to severity of the first flare, COVID-19, and the patient's general condition, we opted for an antibiotic treatment of *Escherichia coli* 0157:H7 while monitoring the parameters of potential hemolytic uremic syndrome development.

The treatment of patients with acute severe ulcerative colitis (ASUC) and coronavirus disease 2019 (COVID-19) presents a clinical challenge. We report on a patient with ASUC and mild COVID-19 acquired during the hospital stay, followed by enterohemorrhagic *Escherichia coli* (EHEC) 0157:H7 infection. To our knowledge, this is the first such case described in the literature.

## Case report

**Figure 1:**
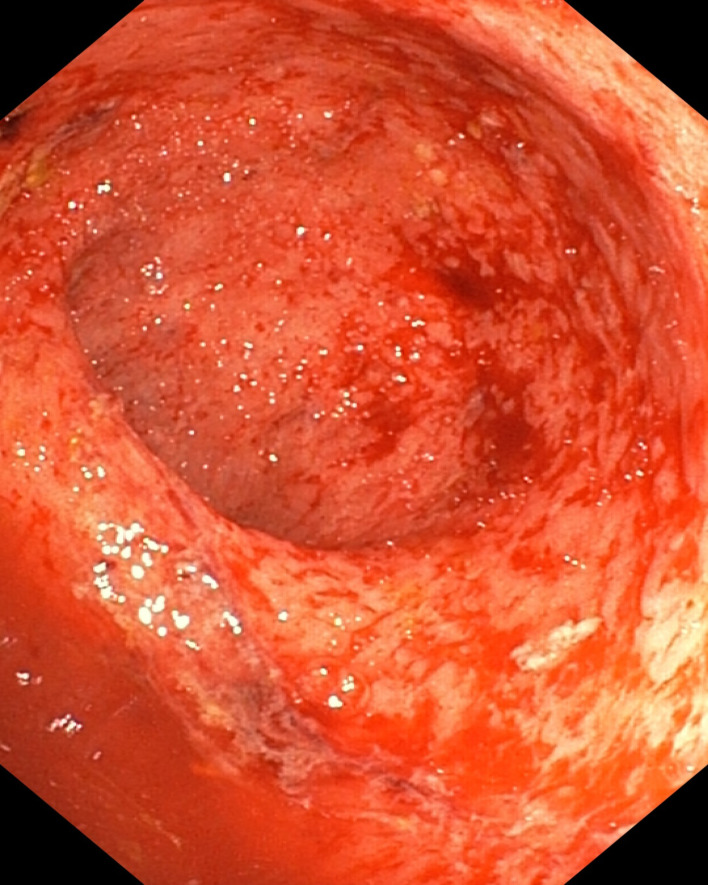
Proctosigmoidoscopy at admission.

A 36-year-old woman, diagnosed with UC in 2019, treated with mesalamine and azathioprine was admitted in 2020 for a relapse. She experienced abdominal cramping with 10 bloody stools a day. Blood pressure was 110/70, heart rate 105/min, respiratory rate 22/min, temperature 38°C, and body mass index 18.7. She lost 3 kg in the two weeks before the admission. Upon admission, C reactive protein was 132 mg/L, leukocytes 10.8 mcL, hemoglobin 12.2 g/L, and albumin 29 g/L. Stool culture for *Clostridioides difficile* toxin was negative. Chest and abdominal x-rays were unremarkable. The nasopharyngeal swab for SARS-CoV-2 was negative. Relapse was classified as severe according to Truelove and Witts severity index. Intravenous methylprednisolone (60 mg IV) was started. Proctosigmoidoscopy showed active disease (Figure 1), Mayo endoscopic subscore was 3, and biopsies excluded *Cytomegalovirus* infection. After initial improvement, the patient’s condition worsened. She developed slight fever with lymphopenia and CRP increase. Nasopharyngeal swab for SARS-CoV-2 (PCR) was repeated and was positive at day 14. She was diagnosed with mild COVID-19. After a few days, she again had 8-10 bloody stools. Interleukin 6 level was high (54.69 pg/mL). Serology test was positive for anti-SARS-CoV-2 IgM, and negative for IgG. She was considered corticosteroid refractory.

Infliximab rescue therapy was administered at a dose of 5 mg/kg. Two weeks after the first dose, due to ASUC, steroid-refractory disease, and proctosigmoidoscopy findings, a second infliximab infusion was administered at a dose of 10 mg/kg. Clinical and laboratory response was achieved. Since the patient had mild COVID-19 without respiratory symptoms, no specific therapy was administered, and she did not develop any post-COVID-19 symptoms. During the hospital stay, the patient was not treated with antibiotics. At discharge, corticosteroid tapering was continued, and no probiotics were administered.

Two weeks after discharge, the patient again complained about a few bloody stools. New CRP was 51 mg/L and fecal calprotectin was over 6000 μg/mg. She was re-admitted. Stool samples tested positive for EHEC 0157:H7. A repeated nasopharyngeal swab for SARS-CoV-2 was negative, while serology tests were positive for anti-SARS-CoV-2 IgM and IgG. Ceftriaxone therapy was administered. A few days after starting antibiotic therapy, stool cultures became negative on EHEC 0157:H7. The patient was discharged from hospital in clinical remission (Table 1).

**Table 1:** Timeline of the disease course and interventions

Date	Relevant medical history	Interventions
November 2020	Patient admitted to hospital for UC relapse presented as ASUC	Corticosteroids therapy
Day 14 of hospitalization	Tested positive for SARS-CoV-2	
Day 19-23 of hospitalization	UC worsened despite therapy	Infliximab rescue therapy
Day 39 of hospitalization (December 2020)	Discharged	
January 2021	Admitted for relapse. Stool tested positive for EHEC 0157:H7^‡^	Antibiotic therapy

*Abbreviations: UC – ulcerative colitis; ASUC – acute severe ulcerative colitis; EHEC 0157:H7 – enterohemorrhagic *Escherichia coli* 0157:H7.

## Discussion

Patients with inflammatory bowel disease (IBD) are not at a higher risk of SARS-CoV-2 infection (1). In addition, the seroprevalence of SARS-CoV-2 in IBD patients treated with biologic therapy is the same as in the general population, and biologic therapy did not affect humoral response in the infected patients (2). IBD patients do not have a worse COVID-19 outcome (3). American Gastroenterological Association guidelines suggest withholding anti-tumor necrosis factor (TNF) agents during viral illness. However, in the case of mild or no COVID-19 symptoms, standard algorithms should be followed, and treatment should be based on bowel inflammation activity (2). SARS-CoV-2-positive patients sometimes have gastrointestinal symptoms, but many also have respiratory symptoms and fever. Preliminary data showed that anti-TNF and anti-IL-12/23 agents did not worsen the disease course, whereas corticosteroids did (4). The PROTECT-ASUC study reported no increased risk of SARS-CoV-2 infection or serious adverse outcomes secondary to COVID-19 in patients with ASUC despite the use of high corticosteroid doses (5).

Since our patient had ASUC and mild COVID-19, it was challenging to decide on the appropriate treatment. Case reports describe continuing anti-TNF agents in patients with COVID-19, but not many patients were started on infliximab for UC within the first 14 days of the infection (6). One case study reported on a 36-year-old patient who was successfully treated for ASUC and COVID-19 pneumonia and one reported on two patients receiving rescue infliximab therapy with no adverse events related to COVID-19 (6,7). Due to the patient's clinical condition and laboratory findings, we considered a first dose of infliximab of 10 mg/kg, but due to lack of evidence for treatment of IBD patients with COVID-19 in the first two weeks, we administered a dose of 5 mg/kg. The patient tolerated the therapy well, but had a partial response (blood in half of her stools), so the second infliximab infusion was administered at a dose of 10 mg/kg. There are limited data on the short-term or long-term efficacy of accelerated infliximab induction therapy for patients with ASUC. A retrospective study and meta-analysis (8) found no association between accelerated infliximab induction regimen and lower rates of colectomy compared with standard induction therapy. However, accelerated infliximab induction regimen could lead to lower early colectomy rates (9).

The second challenge in our case was an enteric infection with EHEC 0157:H7. EHEC 0157:H7 causes a range of diseases, from mild diarrhea to hemolytic uremic syndrome (10). The use of antibiotic treatment in these patients is still debated. Axelard et al (11) detected one case of *E coli* 0157, in a patient with Crohn’s disease, among 577 samples of patients with an IBD flare, and Hanada et al (12) reported one case of EHEC 0157:H7 in stool samples of 1345 patients with an IBD flare.

To the best of our knowledge, our patient was the first to receive rescue biological therapy while being positive to SARS-CoV-2 IgM and with EHEC O157:H7 in induction period. The treatment challenges we faced were biologic therapy administration during active COVID-19, about which little was known at the time, and how to treat EHEC due to the risk of hemolytic uremic syndrome.

In our opinion, a decision on biologic therapy induction for IBD relapse in SARS-CoV-2-positive patients should be made on a case-to-case basis, and driven by the dominant disease. Our patient tested positive for SARS-CoV-2, but actually had mild COVID-19 symptoms. As she had ASUC, we started anti-TNF therapy despite serological tests and the recommendation to delay biological therapy administration for two-weeks. Second, due to the severity of the first flare, COVID-19, and the patient's general condition, we decided to treat EHEC with an antibiotic while monitoring the parameters of potential HUS development.
